# Study and Development of a Fluorescence Based Sensor System for Monitoring Oxygen in Wine Production: The WOW Project

**DOI:** 10.3390/s18041130

**Published:** 2018-04-07

**Authors:** Nicola Trivellin, Diego Barbisan, Denis Badocco, Paolo Pastore, Gaudenzio Meneghesso, Matteo Meneghini, Enrico Zanoni, Giuseppe Belgioioso, Angelo Cenedese

**Affiliations:** 1 LightCube Srl, Viale della Navigazione Interna, 51-35129 Padova, Italy; nicola.trivellin@light-cube.com (N.T.); barbisan@dei.unipd.it (D.B.); 2 Department of Chemical Science, University of Padova, Via Marzolo, 1-35131 Padova, Italy; denis.badocco@unipd.it (D.B.); paolo.pastore@unipd.it (P.P.); 3 Department of Information Engineering, University of Padova, Via Gradenigo, 6/B-35131 Padova, Italy; gaudenzio.meneghesso@unipd.it (G.M.); matteo.meneghini@unipd.it (M.M.); enrico.zanoni@unipd.it (E.Z.); g.belgioioso@tue.nl (G.B.)

**Keywords:** oxygen monitoring, LED, photoluminescence, wine production, fermentation process, process control

## Abstract

The importance of oxygen in the winemaking process is widely known, as it affects the chemical aspects and therefore the organoleptic characteristics of the final product. Hence, it is evident the usefulness of a continuous and real-time measurements of the levels of oxygen in the various stages of the winemaking process, both for monitoring and for control. The WOW project (Deployment of WSAN technology for monitoring Oxygen in Wine products) has focused on the design and the development of an innovative device for monitoring the oxygen levels in wine. This system is based on the use of an optical fiber to measure the luminescent lifetime variation of a reference metal/porphyrin complex, which decays in presence of oxygen. The developed technology results in a high sensitivity and low cost sensor head that can be employed for measuring the dissolved oxygen levels at several points inside a wine fermentation or aging tank. This system can be complemented with dynamic modeling techniques to provide predictive behavior of the nutrient evolution in space and time given few sampled measuring points, for both process monitoring and control purposes. The experimental validation of the technology has been first performed in a controlled laboratory setup to attain calibration and study sensitivity with respect to different photo-luminescent compounds and alcoholic or non-alcoholic solutions, and then in an actual case study during a measurement campaign at a renown Italian winery.

## 1. Introduction

To improve the quality of wine, a crucial step in the production process is fermentation, which is the main topic of past and present research in the wine industry. This research effort is now increasingly focusing on mathematical methodologies to model and optimize the wine fermentation process and novel technologies to support this innovation. Wine fermentation is a very complex bio-chemical process that can be influenced in many ways by the environment and by the addition and subtraction of bio-chemical components, among which oxygen that can be added to the must to play a crucial role for yeast activity [1,2].

In particular, in the wine industry, the term micro-oxygenation [3] refers to the artificial introduction of oxygen through specific devices able to control the dosage. The major advantage of micro-oxygenation is the improvement of wine structure. It is showed experimentally that small amounts of oxygen promote tannic polymerization, favoring a softening of the tannins, a reduction of the extraction and a general improvement of the taste. The injected oxygen also favors the production of larger molecules called polymeric pigments, yielding to color enhancement and an increase in color stability over time. These two benefits, improvement of taste and increase in color, are traditionally obtained by aging the wine in oak barrels for several months [4]. Indeed, although aging in barrels gives advantageous oak aromas to wine, it has been recognized that the greatest benefits of the process are due to the permeability of the walls of the barrique, which allow the diffusion of small quantities of oxygen, thus favoring oxidation reactions that modify the structure and color of wine. The micro-oxygenation therefore is thus frequently presented as an alternative to aging in barriques, with the advantages of a reduced cost, faster maturation and the possibility of scientific control of the process [5].

The major concern of this operation is obviously the oxygen overdose resulting in excess of polymerization, tannic dryness, loss of color, decrement of freshness and development of aldehydes and oxidized aromas: indeed, the fact that there are no clear signs of the transition from the correct dosage to overdose makes this the main risk of micro-oxygenation. Nowadays, in many cellars the control of micro-oxygenation processes is performed through a sensorial analysis of the wine. Wine tasting, in addition to not providing objective results, is carried out only during certain phases of the process and does not allow to prevent undesirable situations caused by the presence of an overdose of oxygen.

Within this context, it was developed the WOW project: deployment of WSAN technology for monitoring Oxygen in Wine products [6], which regards the design of portable sensor devices within a wireless sensor-actuator networks for environmental monitoring, the study of dynamic modeling, and the definition of data analysis methodologies for the continuous and non-invasive oxygen measurement in wine production.

Within the framework of the WOW project, we want to overcome the resistance to innovation of a traditional field, such as that of winemaking by proposing a novel system for the continuous monitoring of dissolved oxygen (DO) concentrations: this would be of great interest in order to retrieve real-time information about the balance between the processes of dissolution and biological consumption of oxygen during the various stages of winemaking, from must to wine aging. On the market there are numerous devices that allow to measure the DO: some are electrochemical, while the most recent use optical technologies, through which the concentration of DO can be measured in a quick, non-intrusive and reliable way. In particular, the purpose of this work is to report the design of a high sensitivity and low cost oxygen sensor that employs an optical fiber system and an especially developed amplification chamber to measure the luminescent lifetime variation of the reference material which is based on a specific metal/porphyrin complex [7]. The final application targets the measurement of the oxygen concentration in liquids, in particular for agro-alimentary application, such as wines and other drinks requiring an enhanced control of the oxygen during development, storage or maturation [8].

The structure of the paper is as follows. After a brief description of the state of the art in methodologies for wine monitoring and fluorescence sensors in Section 2, Section 3 is devoted to the discussion of process monitoring and control in the field of wine production. Then, Section 4 presents the system prototype, entering the details of the adopted LED-based sensor technology and manufacturing and Section 5 describes the validation phase in the laboratory setup and the experimental campaign carried out in a wine industry cellar. Section 6 concludes the paper with a discussion over the results of the system design and of the experimental validation and, finally, proposes some prospective developments to this work.

## 2. State of the Art

In general, molecular oxygen is involved in many chemical and biochemical reactions and its determination is important in different fields, such as environmental and industrial monitoring, biotechnology [9], food industry [10] and medicine [11]. For this reason, oxygen has to be monitored at various concentration levels, from trace analysis (see, e.g., [12,13,14]) to higher concentrations (see, e.g., [15,16]).

### 2.1. Methodologies for Wine Monitoring

To provide an overview of the different methodologies, sensors classification can be accomplished with respect to the signal transduction principles [17] and we can distinguish among:

- optical sensors, based on absorbance, reflectance, luminescence, fluorescence, refractive index, optothermal effect and light scattering;
- electrochemical sensors, including voltammetric and potentiometric devices, amperometric devices and potentiometric solid electrolyte gas sensors;
- electrical sensors including metal oxide and organic semiconductors as well as electrolytic conductivity sensors [18];
- mass sensitive sensors, i.e., piezoelectric devices and those based on surface acoustic waves;
- magnetic sensors (mainly for oxygen) based on paramagnetic gas properties [19];
- thermometric sensors based on the measurement of the heat effect of a specific chemical reaction or adsorption involving the analyte.

In particular, the DO levels during the aging period of the wine, either in barrels or in tanks, are so low that the measurement system requires limit of detection (LoD) close to 10 μg/L at 20 °C, which corresponds to 0.12 % $O_{2}$ (saturation of oxygen in wine at 20 ${}^{\circ }$C and 1 bar is 8.4 mg/L).

Currently, two main technologies for oxygen measurement are available on the market with such capabilities: sensors based on optical technology, commonly called luminescence sensors, and electrochemical sensors. Indeed, as part of the measurement of oxygen in wine matrixes, the most used instrument is the Clark electrode, but the need to continuously monitor DO concentration in solution discouraged its use for the presence of electrolytic solution leaching. Optical sensors are therefore preferred. In the last thirty years optical sensor technology was largely developed and these sensors were miniaturized and cheaper and cheaper. Optical sensors may therefore substitute the electrochemical ones, because they allow in situ, real time, non-destructive measurements. Also, they are more robust than electrochemical ones reducing the need of frequent calibration and membrane replacement.

### 2.2. Fluorescence LED-Based Sensors

Sensors systems based on fluorescence are finding many applications both in laboratory uses and on the field for in situ sensing applications. Fluorescence sensing method is generally preferred over absorbance, since it offers an intrinsic higher sensitivity (see, for example, [20,21,22] for a thorough overview on the subject.

The subject of the present research deals with optical sensors for detecting oxygen. They are based on the quenching, by oxygen, of the luminescence of organometallic complexes embedded in polymeric matrixes. Excitation light is provided by a LED source and a photodiode is employed as detector. When the luminophor absorbs a photon, the energy gain is translated into an excited electron and luminescence is the result of the subsequent electron energy loss between a high energy unstable state and a lower energy state. Luminescence intensity and emission lifetime are dependant on the ambient parameters (temperature, pressure) and chemical composition of the surrounding medium. By measuring the luminescence signal it is, therefore, possible to sense the ambient parameters influencing the luminophor. Since both luminescence intensity and lifetime vary with the ambient parameter the measurement can be carried out in three possible configurations:

**intensity:** luminescent intensity is clearly the most straightforward approach to achieve the measurement of the parameter. Intensity measurement actually has some important drawbacks: (i) both the incident light and luminescence have to be measured, (ii) the luminescence signal needs to be filtered to eliminate the superimposed excitation spectrum, (iii) thermal quenching strongly affects the luminescent intensity, (iv) each luminophor substrate needs a precise calibration to account for thickness, density and transparency variation; (v) calibrated intensity is sensitive to mechanical alterations of the system and optical material degradation [23,24];
**phase shift:** phase shift measurement is more complex with respect to the intensity measurement; it generally requires a lock in amplifier and a precise modulation system for the LED light source. Phase shift presents many advantages being an effective approach to reject noise, making the measurement insensitive to physical properties of the luminophor substrate and limiting the effects of other intensity related system issues. The drawbacks are mainly related to (i) complexity, (ii) sensitivity/bandwidth of the detector and (iii) the luminophor needs to be continuously shined with the sinusoidal signal causing photo-bleaching in particular on sensitive organic compounds [25,26].
**lifetime decay:** lifetime transient can be directly sampled and measured to calculate the recombination exponential mean lifetime, thus directly achieving the value of interest. This approach allows not only to measure the lifetime, but also the intensity and if necessary multi-exponential transients. The drawbacks of the transient measurement are: (i) a higher noise sensitivity, (ii) the necessity to achieve a high speed of the reading system/high sensitivity photodiode feedback topology [27].

## 3. Motivation: Model-Based Control

From the premise given in the introduction, this section aims at providing a motivation to the continuous monitoring of oxygen for wine production with a twofold aim, namely to predict the evolution of the nutrients during the several chemical reactions that occur and to perform a model-based control for micro-oxygenation procedures. In particular, a first step towards this direction is given by the definition of a suitable model for the process under study, that can be complemented with sparse sensor measurements to provide a full picture (in time and space) of the wine production procedure, from must fermentation to wine aging. Indeed, it is to note that while during the first fermentation phase the evolution of the mass of must undergoes tumultuous modifications, driven by strong gradients of temperature and nutrient concentrations, during the final production stages, the reactions are characterized by lower gradients and lower differences between the portion of liquid at the boundary and that in the center of the mass. Thus, throughout the wine production, single points of measurements may result not sufficient to characterize the whole system and suggest the correct action to provide quality improvement to the final product, hence the need for a modeling approach [28].

### 3.1. Reaction-Diffusion PDEs Model

The wine fermentation process dynamics can be modeled through a reaction-diffusion system with nonlinear reaction kinetics that includes spatial diffusion and a heat equation to account for geometrical and thermal effects inside the tank/bioreactor [29,30]. The unknown variables of the system are the space-time dependent functions representing the concentrations of yeast biomass *X*, nitrogen *N*, DO *O*, sugar *S*, ethanol *E*, and temperature *T*. The reaction of these quantities is described by two main relations: the first one, given by Equation (1), expresses the yeast growth, due to cell proliferation thanks to the presence of nitrogen, oxygen, and sugar

(1) $$\begin{array}{c} a_{2}N+a_{3}O+a_{4}S\;\overset{\mu_{1}}{⟶}\;a_{1}X,\; \end{array}$$

the second mechanism, in (2), describes the action of the yeast population that transforms sugar into ethanol and produces heat

(2) $$\begin{array}{c} a_{5}S\;\overset{\mu_{2}}{⟶}\;a_{6}E+a_{7}T, \end{array}$$

where the constants a1,⋯,a7 are suitable coefficients and $\mu_{1}$ and $\mu_{2}$ the reaction rates

(3) $$\begin{array}{c} \mu_{1}\left(X,N,O,S,T\right)\;=\;\left(T-b_{1}\right)\frac{N}{c_{1}+N}\frac{O}{c_{2}+O}\frac{S}{c_{3}+S}X, \end{array}$$

(4) $$\begin{array}{c} \mu_{2}\left(X,S,E,T\right)\;=\;\left(T-b_{2}\right)\frac{S}{c_{4}+S}\frac{c_{5}}{c_{5}+E}X,\; \end{array}$$

with $c_{1},\cdots ,c_{5}$ called the Michaelis-Menten coefficients, which are considered to be constant.

The temperature dependence is assumed to be linear with offsets $b_{1}$ and $b_{2}$ representing stagnation temperatures. For the nutrients *N*, *O* and *S*, the Michaelis-Menten terms ($\frac{N}{c_{1}+N}$, etc.) lead to a saturation of the reaction rate for high concentrations. Moreover, they lead to no reaction if any nutrient is consumed. DO *O* and sugar *S* are considered as additional nutrients, because the yeast modifies its metabolism from aerobic to anaerobic in the absence of oxygen and stops cell division. The inhibition property of ethanol for the sugar consumption is taken into account by a term of the form $\frac{c_{5}}{c_{5}+E}$, which yields a lower reaction rate at high ethanol concentrations.

The resulting partial differential equations that also include diffusion are as follows,

(5) $$\begin{array}{c} \frac{\partial X}{\partial t}-\sigma_{1}\Delta X=a_{1}\mu_{1}\left(X,N,O,S,T\right)-\Phi \left(E\right)X, \end{array}$$

(6) $$\begin{array}{c} \frac{\partial N}{\partial t}-\sigma_{2}\Delta N=-a_{2}\mu_{1}\left(X,N,O,S,T\right), \end{array}$$

(7) $$\begin{array}{c} \frac{\partial O}{\partial t}-\sigma_{3}\Delta O=-a_{3}\mu_{1}\left(X,N,O,S,T\right), \end{array}$$

(8) $$\begin{array}{c} \frac{\partial S}{\partial t}-\sigma_{4}\Delta S=-a_{4}-a_{5}\mu_{2}\left(X,S,E,T\right), \end{array}$$

(9) $$\begin{array}{c} \frac{\partial E}{\partial t}-\sigma_{5}\Delta E=a_{6}\mu_{2}\left(X,S,E,T\right), \end{array}$$

(10) $$\begin{array}{c} \frac{\partial T}{\partial t}-\sigma_{6}\Delta T=a_{7}\left(X,S,E,T\right), \end{array}$$

where the diffusivity coefficients $\sigma_{1},\cdots ,\sigma_{6}$ are positive constants [31,32]. Equations (5)–(10) are defined in the space-time cylinder $Q=\Omega \times (0,t_{f}),$ where $\Omega \subset R^{3}$ is a bounded domain that represents the interior of the tank, and $t_{f}$ is the final time of the fermentation process. The term Φ(E)X in (5) models the dying of the yeast population at the end of the fermentation process due to a toxic concentration of ethanol.

Interestingly, the heating exchanges through the boundary of the tank Γ=∂Ω can be regulated by a cooling/heating system made of tubes in contact with a section $\Gamma_{2}$ of the side walls of the bioreactor. The remaining surface of the tank boundary $\Gamma_{1}$, composed by the upper and bottom walls and part of side walls, exchanges heat directly with the external environment of the winery. These two mechanisms are included in the model exploiting the following Robin-type boundary conditions:

(11) $$\sigma_{6}\frac{\partial T}{\partial n}=\left\{\begin{array}{cc} \tau_{air}\left(T_{ext}-T\right) & \Gamma_{1}\times \left(0,t_{f}\right)\\ \tau_{water}\left(T_{u}-T\right) & \Gamma_{2}\times \left(0,t_{f}\right) \end{array}\right.$$

where $T_{ext}$ and *u* are the temperature of the environment and the temperature of the water in the cooling/heating system, $\tau_{air}$ and $\tau_{water}$ represent the thermal conductivity of the tank walls exposed to air and water, respectively.

### 3.2. Boundary Temperature Control

Based on this model, an example of process control with respect to must fermentation is discussed hereafter, where it is highlighted the necessity of continuous monitoring of the oxygen and the other nutrients to opportunely act on the controller gains.

The model (5)–(10) with boundary condition (11) makes possible to simulate the temporal and spacial dynamics of the main components of the must during a fermentation process. In order to perform a simulation it becomes necessary to set:

- the values of model parameters, i.e., yield coefficients $a_{i}$, Michaelis-Menten constants $c_{i}$, diffusivity coefficients $\sigma_{i}$ and thermal conductivities $\tau_{*}$,
- the initial conditions of the unknown variable, i.e $X_{0}$, $N_{0}$, $O_{0}$, $S_{0}$, $E_{0}$, $T_{0}$.

Model parameters change from system to system and depend mainly on materials, shape and size of the tank/bioreactor. They can be identified exploiting data from real experiments and standard identification techniques. On the other hand, the initial condition of the unknown variables correspond to the initial concentrations of yeast biomass, nutrients, reactions products and the temperature of the must. Their values can be easily measured just before the beginning of the process. All the following results refer to a bioreactor system whose parameters and initial conditions have been taken from the literature. Furthermore, the temperature of the cellar and the temperature of the cooling water have been assumed constant all over the process, i.e., $T_{ext}=T_{u}=$ 16 ${}^{\circ }$C.

**Figure 1:**
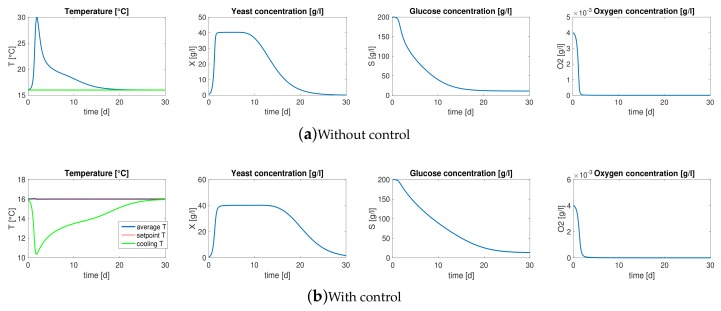
Model dynamics. (**a**) Average behaviors of must main components during 30 days of fermentation, the temperature of the cellar and the temperature of the cooling water are constant all over the process, i.e., *T_ext_* = *T_u_* = 16 °C. (**b**) Average dynamics of the bioreactor where a PID controller with gain scheduling drives the cooling/heating system in order to keep the temperature at the optimal value 16 °C.

Figure 1a represents the average behaviors of must main components during 30 days of fermentation of the system described above. It is remarkable to note that the average temperature inside the bioreactor increases drastically during the first days of the process reaching 30 ${}^{\circ }$C: in most of the cases, this temperature could be lethal for some components of the must and thus detrimental for the organoleptic properties of the final product.

Exploiting the cooling/heating system and a simple control architecture it is possible to keep the bioreactor within a safety range of temperature. In particular, in this scenario a PID controller with gain scheduling is chosen, which is one of the most popular control techniques in industrial process control [33]. To this aim, we assume to be equipped with a system of on-line measurement for the average temperature $T_{a}$, the average concentration of yeast $X_{a}$ and DO $O_{a}$ in the must, as that proposed in this work in Section 4: the strong non-linearity of the dynamics makes mandatory that the controller gains are tuned according to the current working point of the system and hence it arises the need to measure $X_{a}$ and $O_{a}$ on-line.

Figure 1b shows the average dynamics of the bioreactor where the controller drives the cooling/heating system in order to keep the temperature around an optimal value 16 ${}^{\circ }$C. Note that the average temperature $T_{a}$ (blue line) of the must remains within the range of ±0.5 ${}^{\circ }$C from the setpoint (red line) during the entire process. While the temperature of the cellar remains constant, under the control action the temperature of the cooling water $T_{u}$ decreases drastically during the first days in order to contrast the increase of temperature brought by the exponential growth of the yeast and then settles at a steady state.

**Figure 2:**
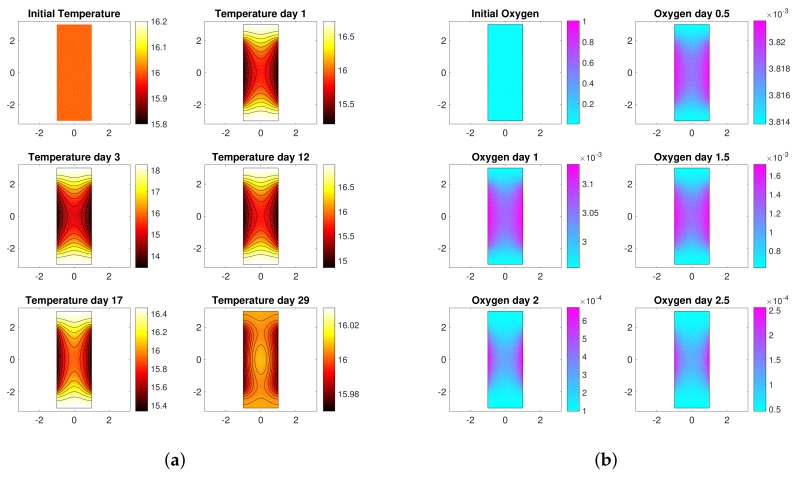
Model dynamics. (**a**) Temperature distributions throughout a section of the bioreactor during some phases of the fermentation process. (**b**) Oxygen gradients throughout a section of the bioreactor during some phases of the fermentation process.

Since the cooling action of the water operates only at the boundary, the temperature of the must does not result uniform throughout the bioreactor (Figure 2a shows the distributions of temperature inside the tank during the process). As a consequence, gradients of concentration of the nutrients arise throughout the bioreactor (see also [28,34]). Figure 2b represents the concentration gradients of oxygen during some phases of the process. Note that the concentration of oxygen is always higher close to the side walls, where the temperature is lower and the reactions are slower. After just 2 days oxygen is almost over and the concentration values decrease by one order of magnitude, as it can be seen also from Figure 1b.

## 4. Monitoring System Design

### 4.1. Luminophor Choice

In the WOW project, attention has been focused on the luminescence-lifetime-based sensors, because they are more promising to build low cost industrial sensors. The Stern-Volmer model describes the dynamic quenching, and oxygen concentration (expressed in %) may be obtained from luminescence quenching according to:

(12) $$\frac{\tau_{0}}{\tau }=1+K_{SV}\;\cdot \;\%O_{2}$$

where τ is the luminescence excited-state lifetime of the luminophor, $\tau_{0}$ is the same parameter in the absence of oxygen, the Stern Volmer constant $K_{SV}$ is proportional to the luminophor lifetime in the absence of oxygen ($\tau_{0}$), oxygen diffusion coefficient in the polymeric membrane ($D_{O_{2}}$), oxygen solubility into the membrane ($s_{O_{2}}$), and pressure (*P*): $K_{SV}\propto \tau_{0}D_{O_{2}}s_{O_{2}}P$. Luminophor with various lifetimes in the absence of oxygen have been tested in order to optimize the sensor analytical performance, namely [35,36,37,38,39]:

- ruthenium tris-(4,7-diphenyl-1,10-phenanthroline) bis(octylsulphate) (Ru(dpp)OS, $\tau_{0}=6$ μs)
- 5,10,15,20-Tetrakisphenyl-21H,23H-porphine platinum(II) (PtTPP, $\tau_{0}=50$ μs)
- 5,10,15,20-Tetrakis(pentafluorophenyl)-21H,23H-porphine platinum(II) (PtTFPP, $\tau_{0}=70$ μs)
- 5,10,15,20-Tetrakis(pentafluorophenyl)-21H,23H-porphine palladium(II) (PdTFPP, $\tau_{0}=850$ μs)
- 2,3,7,8,12,13,17,18-Octaethyl-21H,23H-porphine palladium(II) (PdOEP, $\tau_{0}=990$ μs)

The choice of the luminophor was made on the basis of the luminescence lifetime. Too short a lifetime complicates the sensor electronics as a short time has to be measured, while too long a lifetime does not allow to get sensible SNR values. The ideal trade-off was achieved by using the PtTFPP/PSF supported on a Mylar^®^ substrate. A spin-coating deposition was used. This membrane reaches low LoD, down to 0.1% in wine at 20 ${}^{\circ }$C and 1 bar with respect to saturation.

### 4.2. Sensor Structure and Description

The direct transient measurement has been chosen as the operating principle of the sensor in order to develop a more complete, customizable system able to acquire the complete transient and then focus on the signal elaboration [40]. Moreover, the ultra-short excitation pulses required to achieve the lifetime measurement imply that the photo-bleaching is minimal with respect to other measurement approaches. Sensors embedding PtTFPP/PSF were continuously tested 24 h a day for one month. If that test is carried at room temperature (20 ${}^{\circ }$C), the luminescence decrease is close to 5%. After two years we estimate that the sensor is still functional, and the emission intensity decreases to 30% owing to the SNR reduction. The mechanical sensing structure of the detector is based on local light reflection, avoiding the use of optical fiber and waveguide to improve the luminescence signal intensity. The exciting source and the sensing electronics has been designed to be placed on the same side, directly in close contact with the luminescent substrate thus allowing the direct contact with the liquid to be measured. The reflectivity of the luminophor has been optimized with the use of a high reflectance coverage substrate permeable to the oxygen dissolved in the liquid.

The sensor structure is based on the exciting element, the luminophor and the detector. The exciting source is composed of 4 low power LEDs emitting in the UVA range (395 nm), the driving current is 20 mA (a constant current is necessary to achieve a stable irradiance and excitation wavelength as presented in [41]) to achieve a total optical power of 20 mW. By using 4 small size LEDs it has been possible to optimize the optical structure and achieve a more effective excitation. Using multiple sources located around the detectors allows the reduction of the average light travel distance between the source, the luminophor and the detector.

**Figure 3:**
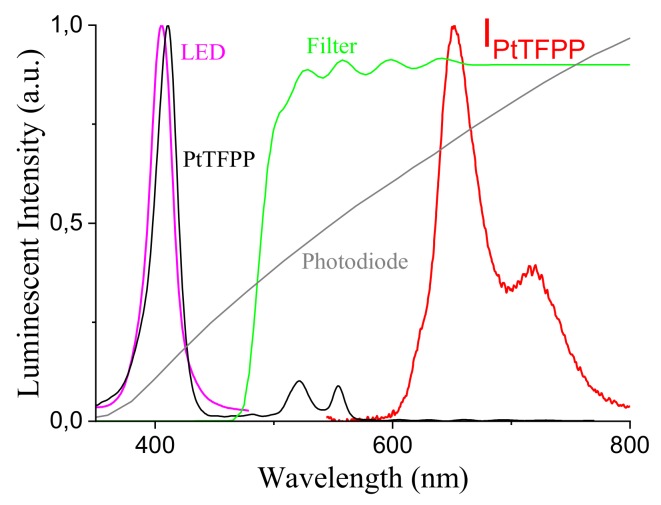
LED emission spectrum, PD response, luminophor absorption and emission, and optical filter spectral transmission.

The luminophor is based on a specifically engineered PtTFPP/PSF membrane supported on a Mylar^®^ substrate. The Mylar^®^ support compared to the glass one facilitates the construction of the sensor and allows to improve the geometry of the sensor and therefore the overall emission yield. In order to improve the signal to noise ratio (SNR) a PVDF (polyvinylidene fluoride) film was deposited on the sensing membrane, since the signal acquired from the photodiode is enhanced by the reflection given by PVDF layer. The most important characteristic of this kind of optical sensors is that the membrane may degrade (even though for a lower extent with respect to Clark electrodes, for instance) but the lifetime is always the same so that the measurement remains correct. The absorption peak of the luminophor is centered at 400 nm, while the luminescent emission spectrum is broader and peaked at around 650 nm. The detector is based on a commercial Si photodiode, with a broad spectral response peaked at 850 nm, the photosensitive area is 1×1 mm^2^, allowing a good tradeoff between a short switching time (typical of 5 ns) and a good sensitivity of 0.65 A/W, thus allowing for high speed signal sampling and a sufficient sensitivity to the low level signal emitted from the luminescence. The main complex design task is related to the development of an high speed transimpendance amplifier for the sensor, requiring a specific guard ring to enhance noise reduction and improve the gain bandwidth product to a value of 500 MHz [42,43]. The photodiode has been filtered to reject UV-blue wavelength by means of a LEE filter film (sample Medium Yellow 010). In Figure 3 we report the relative spectrum of the LED emission, the PD response, the luminophor absorption and emission, and the optical filter spectral transmissivity.

**Figure 4:**
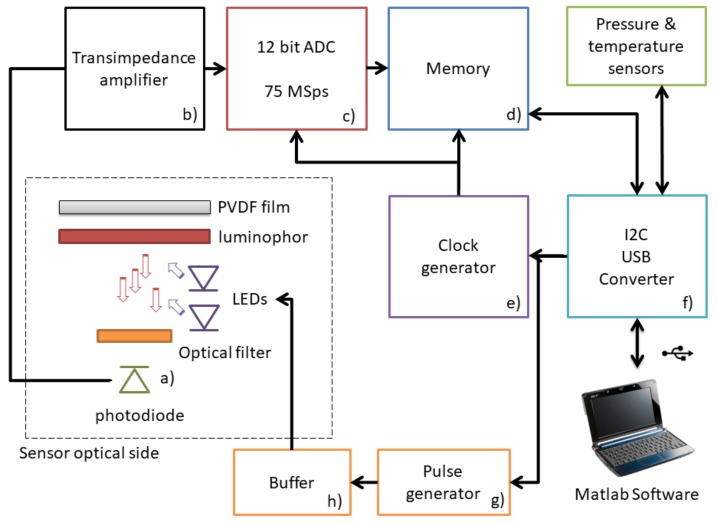
Block diagram of the electronic structure of the designed system. The Photodiode and the Transimpedence amplifier constitute the analog front end, while ADC, memory and I2C-USB interface are the digital section of the sensor.

The combination of the optical structure and the high bandwidth amplifier has entailed the substantial advantage of being able to use a traditional p-i-n photodiode, instead of an avalanche photodiode. The avalanche photodiode, exploiting the effect of amplification of the number of carriers, allows to detect even very weak signals, but its non linear response with the light intensity makes it difficult to read and compensate for light transients; furthermore, the cost of an avalanche photodiode is much higher than a traditional photodiode, and a rather high voltage (between 50 and 200 volts) is required to generate the avalanche effect. The development of a standard Si-based system has been the core element of this work in order to achieve a compact, low cost in situ sensor. We estimate that the electronic cost of the developed sensor should be in the order of 30 to 50 US Dollars (USD) at the time of the present article according to manufacturing volumes; the price of commercially available oxygen sensors with similar characteristics is in the order of 500 to 1000 USD, but it accounts also for ancillary costs (commercialization, retail, etc.). As a reference the cost comparison between an avalanche and a standard Si photodiode is 50–100 USD versus 0.5 USD. The block diagram of the sensor is reported in Figure 4. The photodiode (Si-based with a sensing area of 1×1 mm^2^, a 380–1100 nm spectral response and a typical switching time of 5 ns) is not biased, and the generated current is amplified by means of the high bandwidth transimpedance amplifier (an ultra low bias Op Amp with a 500 MHz Gain Bandwidth product; (b) in Figure 4). Generated electrical signal has been then sampled by means of an high speed Analog to Digital Converter (ADC) (a 12 bit ADC with a maximum conversion rate of 75 MSps with a 3 V reference signal; (c) in Figure 4) at a sampling rate of 75 MSps and stored onto a digital memory (a FIFO (first in first out) memory with a capacity of 4096 18-Bit data, at a maximum frequency of 133 MHz; (d) in Figure 4), and the synchronization between ADC and memory is guaranteed by a quartz-based clock generator (a CMOS programmable clock generator, (e) in Figure 4) that produces a precise square signal. After the acquisition, the memory can be read by means of an Inter Integrated Circuit–Universal Serial Bus (I2C to USB ) interface chip (an I/O Expander with serial interface; (f) in Figure 4). Finally, the electronic board interfaces with a specific MATLAB^®^ software (MathWorks, Natick, MA, USA) running on a personal computer.

The converter controls both the clock generator, the memory start-up sequence and triggers the pulse generator; it also reads the memory and sends the output data collected from the memory to the PC. The pulse generator (a monostable pulse generator, with a pulse width range between 1 μs and 30 s; (g) in Figure 4) drives the LEDs by means of a current buffer (realized by means of a current feedback polarized dual high speed metal-oxide-semiconductor field-effect transistor (MOSFET) driver; (h) in Figure 4), which precisely regulates the LEDs current to 20 mA per chip for a pulse duration of 100 μs.

In close proximity to the optical sensor, the sensor body is equipped with a pressure sensor. The pressure sensor has a silicone enclosure and has a maximum measure value of 30 bar. A temperature sensor completes the prototype: it is placed on the back of the body, directly connected to the steel backplate to improve the thermal connection with the external liquid. Both the pressure and temperature sensors communicate by means of I2C protocol, conveyed to USB. The two added sensors are necessary to allow the calibration of the oxygen chemical balance between the sensing substrate and the environment.

**Figure 5:**
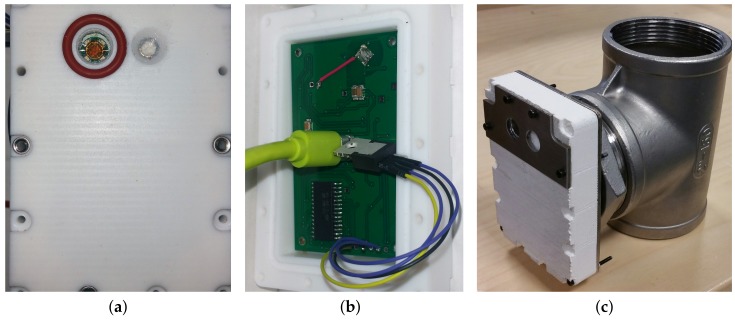
Sensor head prototype. (**a**) Front of the sensor with the photodiode window (with silicone gasket) on the left and pressure sensor on the right. (**b**) Back of the sensor without the stainless steel cover: printed circuit board (PCB) digitalization board (green), USB connection (light green) and wired temperature sensor are visible (TO220 package). (**c**) Sensor prototype on the tubular support. The sensor head size including the PTFE case and stainless steel flanges is: 87×61×20 mm^3^.

The three panels of Figure 5 show the final sensor prototype, highlighting the photodiode window (Figure 5a) and part of the electronic board (Figure 5b), together with the final accommodation of the sensor head on the tubular structure (Figure 5c).

## 5. Experimental Validation

### 5.1. Laboratory Experimental Details and Results

#### 5.1.1. Reagents

Tetrahydrofuran (THF), polysulfone (PSF), MN:16.000, MW: 35,000, were obtained from Aldrich Products. 5,10,15,20-Tetrakis(pentafluorophenyl)-21H,23H-porphine Platinum(II) (PtTFPP) was obtained from Frontier Scientific. Ultrapure water was obtained with a Millipore Plus System (Milan, Italy, resistivity 18.2 MΩ cm^−1^). Immun-Blot^®^ PVDF Membrane 0.2 μm pore size (BIO-RAD Laboratories Srl, Milano, Italy).

#### 5.1.2. Membrane Preparation

The membrane was prepared as follows: 1 mg of PtFTPP was dissolved in 1 mL of a 0.1 g/mL PSF solution in THF. 50 μL of the final solution were deposited by spin coating at 35 r.p.s. on a Mylar^®^ support (GECAM Srl, Minerbio, Bologna, Italy). The membrane was conditioned at 90 ${}^{\circ }$C for 24 h and then calibrated with known $N_{2}/O_{2}$ gas mixture into the chosen solution (water, water/ethanol 12% *v*/*v*, white wine, red wine). The thickness of the sensing layers were measured with a spectroscopic ellipsometer mod. Alpha-SE^®^ (J.A. Woollam Co. Inc., Lincoln NE, USA). The layer thickness is close to 2.8 μm. The response time in air ($t_{90}$), starting from $N_{2}$ is 5 s, while the response time in wine is longer (close to 50 s) owing to a slower diffusion of $O_{2}$ through the PVDF scattering membrane.

#### 5.1.3. Sensor Performances

In this section, we report on the performance measurements of the sensor basic sub blocks and complete structure. The measurements capabilities of the system have been evaluated, by analyzing: (i) the SNR of sensor analog front end and digital conversion circuit, (ii) the sensor calibration procedure, (iii) the oxygen conversion algorithm. Gas mixtures were flown via Alicat Scientific mass flow controllers (code MC-100-SCC-D, calibrated with pure $O_{2}$ and $N_{2}$ into the sensor cell and mixed via a homemade mixer.

The testing and validation of the sensor have been carried out by means of two phases: in the first phase the device has been placed in a controlled gaseous environment where nitrogen and oxygen are fluxed by means of digitally controlled gas valves, the chamber is equipped with a small vent valve and kept at a positive relative pressure to avoid the influence of the atmospheric gaseous components. In the second phase the sensor was submerged in water where oxygen was chemically reduced by bisulfite to guarantee a zero concentration.

#### 5.1.4. Digitalization of the Luminescence Signal

**Figure 6:**
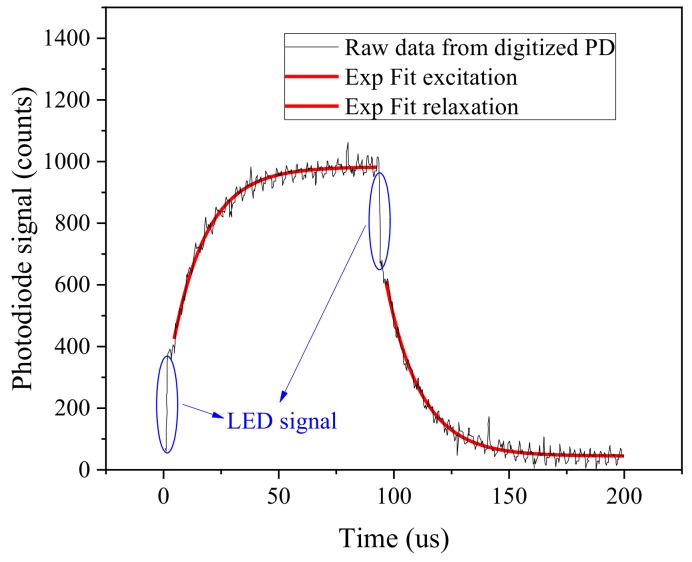
Digital converted photodiode raw data response of a complete measurement transient, PtTFPP/PSF luminophor. Exponential fitting is superimposed (red line) and residual LED signal are highlighted (blue circles).

Figure 6 reports the kinetic transient during a sampling transient at an ambient temperature of 25 ${}^{\circ }$C, pressure of 1 bar, a 21% oxygen concentration is expected since the measurement is taken in air at 25 m altitude. The sensor is equipped with a PtTFPP/PSF membrane on Mylar^®^. The excitation and relaxation transients as detected and digitalized by the photodiode are visible.

It is possible to notice that, although the LED signal has been suppressed by the filter it is still detectable from the photodiode, and it is reported as a fast transient (LED switching kinetic is measured in approximately 50 ns) at the beginning of excitation (close to 0 s) and at the beginning of relaxation (100 μs). Since the intensity of the LEDs is well controlled by the current buffer and not causing any sensible self heating of the LED it is not affecting the measurement carried out during LED excitation. To suppress any possible effect of the LED signal, the measurement can be collected only during photoluminescence relaxation phase. The photoluminescence decay $y_{PD}$ can be well fitted with a single lifetime exponential, where the first 10 μs are excluded from the fitted curve. The fitting equation reads as follows:

(13) $$y_{PD}=\alpha +\beta \times e^{\gamma t}$$

with α,β,γ fitting parameters; the value of τ can be retrieved as $\tau =-{\left(\gamma \times 10^{-6}\right)}^{-1}$. Results indicate that both excitation and relaxation have similar lifetime constant, 14.83 μs and 14.98 μs respectively for the excitation and relaxation kinetics. During the development phase of the sensor different pulse duration have been tested. Reducing the pulse duration below 100 μs has the effect of reducing the luminescence signal, while further increasing the pulse duration does not have a significant effect on measurement performances, thus causing photo-bleaching and reducing the sensor reliability. The hardware amplification of the transimpedance has been chosen to achieve a maximum signal intensity of approximately one quarter of the entire dynamic range at sea level atmospheric conditions and room temperature, thus allowing the correct measurement for higher signal intensities as a function of oxygen concentrations and temperature. The noise of the sensor has also been characterized, a maximum theoretical SNR of 34.5 dB, and a 28.4 dB at a quarter of the dynamic range. The SNR, although limited, is still sufficient to achieve good exponential fitting of the transients allowing a fit determination coefficient $R^{2}$ higher than 0.97.

#### 5.1.5. Sensor Characterization in Controlled Environment

**Figure 7:**
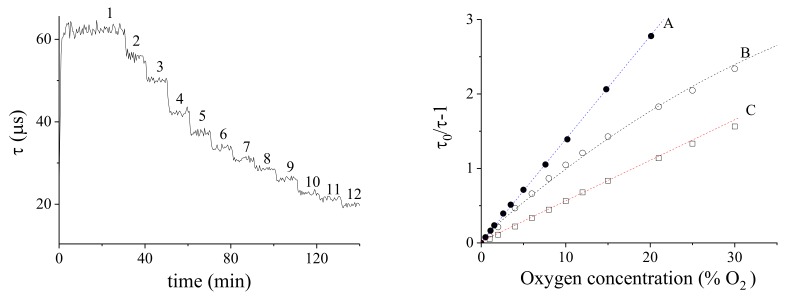
(**a**) Luminescence-lifetime of a PtTFPP/PSF membrane versus time, at various oxygen/nitrogen concentrations in 12% ethanol solution; 1-12 labels correspond to the following $\%O_{2}$ values: 0, 1, 2, 4, 6, 8, 10, 12, 15, 21, 25, 30. (**b**) Stern-Volmer calibration plots; A: in air (membrane supported on glass); B: in air (membrane supported on Mylar^®^); C: in 12% ethanol solution (membrane supported on Mylar^®^). All calibrations were obtained at 20 ${}^{\circ }$C and 1 bar.

The sensor has then been characterized at different oxygen levels by inflating a liquid container with pre-regulated gaseous mixtures, results are described in the following. The Figure 7a plot represents the measurement of the luminescence lifetime of various oxygen/nitrogen mixtures inflated in a 12% ethanol solution versus time, by using a PtTFPP/PSF membrane on Mylar^®^. The membrane peak lifetime is reached in the absence of oxygen, while it decreases with increasing oxygen concentration.

#### 5.1.6. Sensor Calibration

The sensor calibrations was made with oxygen concentration ranging from 0% to 30%. Figure 7b reports the Stern-Volmer plot relative to (A) calibration in air of a membrane PtTFPP/PSF supported on glass, (B) calibration in air of a membrane PtTFPP/PSF supported on Mylar^®^ and (C) calibration in a 12% ethanol solution of a membrane PtTFPP/PSF supported on Mylar^®^. The linear regression slope is $K_{SV}$. The non-linearity observed in (B) was due to the intermixing of the PSF and Mylar^®^ produced by the THF solvent. The linear, low slope calibration of (C) is probably a consequence of the solubilization of ethanol inside the membrane (see later).

The fitting equation comes from the Stern-Volmer relation (12) and is the following

(14) $$\frac{\tau_{0}}{\tau }-1=a\left(s_{a}\right)+b\left(s_{b}\right)\%O_{2}$$

where *a* and *b* are intercept and slope, respectively, and $s_{a}$ and $s_{b}$ are their errors. In this equation $a(s_{a})$ should be statistically 0 as experimentally obtained in the cases A and C in Figure 7b. We obtained:

A: in air (membrane supported on glass):
  $\frac{\tau_{0}}{\tau }-1=0.006\left(0.009\right)+0.12\left(0.001\right)\%O_{2}$
B: in air (membrane supported on Mylar^®^):
  $\frac{\tau_{0}}{\tau }-1=0.06\left(0.004\right)+0.101\left(0.003\right)\%O_{2}-0.00076\left(0.00007\right)\%O_{2}^{2}$
C: in 12% ethanol solution (membrane supported on Mylar^®^):
  $\frac{\tau_{0}}{\tau }-1=-0.003\left(0.003\right)+0.056\left(0.003\right)\%O_{2}$

The calibration sensitivity is proportional to the solubility of $O_{2}$ within the membrane: the permeation and the subsequent adsorption/absorption of ethanol reduces this parameter, which reflects in a slope decrease (Figure 7a, from case A-B to C).

**Figure 8:**
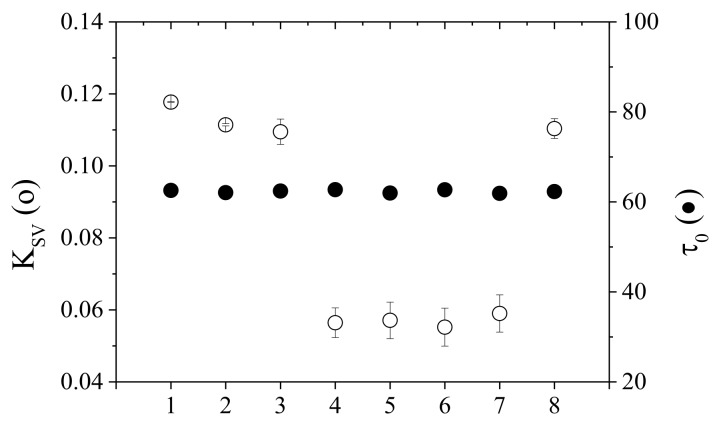
Life time $\tau_{0}$ in the absence of oxygen and $K_{SV}\left(T\right)$ calculated in various conditions at 20 ${}^{\circ }$C; x-axis labels refer to (1) just prepared membrane in air; (2) in air after heat treatment at 90 °C for 24 h; (3) in water; (4) in white wine; (5) in red wine; (6) in 12% ethanol solution; (7) in water after (6); (8) in water after heat treatment at 90 °C for 24 h.

Figure 8 shows the lifetimes in the absence of oxygen and the calibration sensitivity (SV constant) in various calibration environments at 20 ${}^{\circ }$C and 1 bar. In particular, the membrane was calibrated in air after preparation (1), calibration in air after heat treatment of 24 h at 90 ${}^{\circ }$C (2), calibration in water (3), calibration in white wine (4), calibration in red wine (5), calibration in 12% ethanol solution (6), calibration in water after 3 days of immersion (7) and calibration in air after heat treatment for 24 h at 90 ${}^{\circ }$C (8) after use. The error bars represent the error $\pm s_{K_{SV}}$ computed on three independently prepared membranes.

The lifetime value in the absence of oxygen ($\tau_{0}$) remains constant (62.4 ± 0.4 s) in all cases allowing to exclude the presence of any interfering species in wine. The calibration sensitivity is halved by passing from air and water to wine. Ethanol remains adsorbed in the membrane but the membrane performance is completely recovered after the thermal treatment. It results from these experiments that the calibrations performed in milliQ water, in red wine, in white wine and in wine-like are practically identical (see Figure 8, cases (4)-(5)-(6)-(7)). This indicates that ethanol does not modify the membrane structure and that the sensitivity decrease depends on the oxygen solubility in the presence of ethanol inside the membrane.

Each membrane was calibrated before use by means of lifetime measurements. The lifetime value used in model (12) is the median value of 10 consecutive measurements in thermodynamic equilibrium conditions. The value of the LoD is 0.05% $O_{2}$ for the membrane if calibrated in air and goes to 0.1% $O_{2}$ when the calibration is made in wine matrices, due to the loss of calibration sensitivity. The calculation of the LoD is based on [44]. Also, the repeatability of the measurements was monitored by repeating the calibration one year after membrane preparation. A small loss in the precision of the measurements, due to a 30% reduction of the SNR, was observed, leading to an LoD increase. More precisely, a signal decrease of 30% does not alter the lifetime but increases the LoD value from 0.1% $O_{2}$ to 0.15% $O_{2}$ at 20 ${}^{\circ }$C [44]. The error relative to a 10% $O_{2}$ is close to 0.2% [45].

#### 5.1.7. Algorithm for Oxygen Determination

The algorithm for calculating the oxygen concentration expressed in % was derived from the Stern-Volmer Equation (12):

(15) $$\%O_{2}=\left(\frac{\tau_{0}\left(T\right)}{\tau }-1\right)\frac{1000}{K_{SV}\left(T\right)\;\cdot \;P}$$

**Figure 9:**
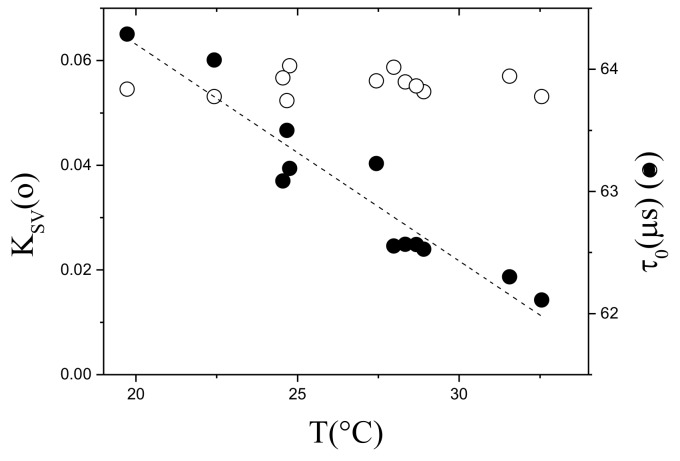
Calibration of $\tau_{0}\left(T\right)$ and $K_{SV}\left(T\right)$ with *T*. PtFTPP-PSF membrane supported on Mylar^®^ made in 12% ethanol.

In this equation the parameters to be measured in time are τ, *T* and the experimental *P* in mbar, respectively. $\tau_{0}\left(T\right)$ and $K_{SV}\left(T\right)$ must be calibrated with temperature in 12% ethanol. Figure 9 shows the calibration between 18 ${}^{\circ }$C and 30 ${}^{\circ }$C. The lifetime decreases with increasing temperature while $K_{SV}\left(T\right)$ remains almost constant because the decrease of $\tau_{0}\left(T\right)$ is compensated by the increase of oxygen permeability with *T* in the membrane.

### 5.2. In Situ Experimental Details and Results

#### 5.2.1. Experimental Details

The oxygen sensor has also been tested on the field and this experimental campaign has been carried out at an international renowned winery Azienda Agricola Monteci [46] located in the Valpolicella region (Verona, Italy). The experiment has been carried out onto a red wine tank of the variety Amarone della Valpolicella, the tank is a stainless steel container with a capacity of 600 hL and an height of 4.9 m.

**Figure 10:**
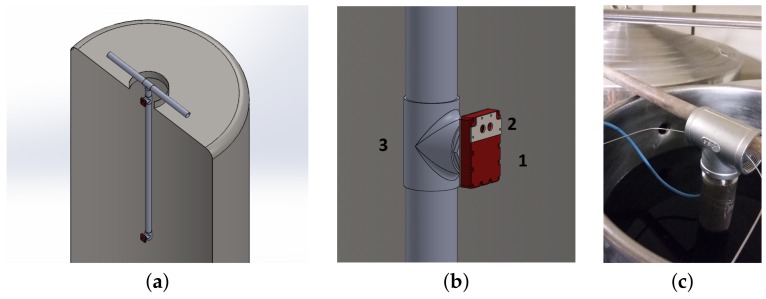
Measurement system. (**a**) Multisensor structure. (**b**) Sensor body (1,2) mounted onto the support tube (3). (**c**) The measurement tube inserted into the Amarone Wine during the in situ measurement.

To recall the main design features, the oxygen sensor has been encased into a food certified Polytetrafluoroethylene (PTFE)/stainless steel casing. The case is equipped with three windows: the first to place the luminophor in contact with the liquid to be measured, the second for the pressure sensor, the third (located on the back) allows the mechanical connection with the support system and the wire electrical connection. The encased structure of the sensor board and the PTFE case is in the following referred as sensor body (see Figure 10b). Since these measurements have local validity, providing information near the sensor’s sensitive membrane, it is necessary to use more sensor bodies to sample at different zones inside the container, as also suggested in the modeling section analysis. In this case, the use of two sensors at a different depth of the tank, is considered sufficient to monitor the state of the entire wine mass, and can be complemented with a model of the process to provide a more thorough view. Thus, the measurement system has been equipped with two identical oxygen sensor bodies positioned along a stainless steel tube with a diameter of 50.8 mm by means of threaded T tube junctions (Figure 10a). The tubular structure hooks onto the mouthpiece at the top of the tank, thanks to its characteristic shape (Figure 10c), and goes inside it without touching the bottom or the walls. The function of this structure is twofold: on the one hand, it maintains the sensors at a fixed position inside the tank at a depth of 0.1 m and 2.1 m respectively, while on the other it allows the cabling necessary for the continuous signal acquisition to be processed in real-time.

The tube has been placed into the wine tank immediately after a wine aeration, i.e., a wine decanting phase where the liquid flow is purposely turbulent to incorporate oxygen into the wine. This procedure should allow the tracking of the oxygen kinetic during the consequent phase of oxygen reduction.

#### 5.2.2. Results

**Figure 11:**
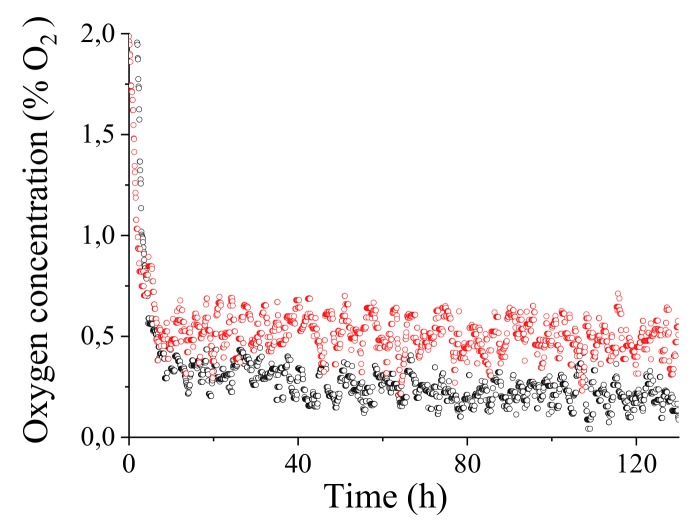
$\%O_{2}$ measured every 5 min by two sensors placed in an Amarone tank at 10 cm and 210 cm from the overflow surface of the wine. Red: depth 10 cm (1040 bar, 20 °C) average concentration $O_{2}=0.50\%$. Black: depth 210 cm (1220 bar, 19 °C) average concentration $O_{2}=0.23\%$.

Figure 11 shows the oxygen concentration resulted from a 5 days continuous monitoring in the Amarone tank. Red and black symbols refers to two sensors placed at 10 cm (red) and at 210 cm (black) of depth from the surface. It is to note that by resorting to (15), the oxygen concentration directly depends on temperature (through parameters $\tau_{0}\left(T\right)$ and $K_{SV}\left(T\right)$) and pressure: the compensation process is therefore implicit in the calibration procedure by exploiting the in situ concurrent sensor measurements of temperature and pressure at a depth of 10 cm (1040 bar, 20 ${}^{\circ }$C) and a depth of 210 cm (1220 bar, 19 ${}^{\circ }$C).

The average oxygen concentration obtained are quite low, 0.23% and 0.50% with a standard deviation of 0.074 and 0.089, measured with the sensor deep inside the tank and on the surface, respectively. The oxygen concentration after an initial conditioning remained on average constant throughout the sampling time of 5 days.

## 6. Discussion and Conclusions

For a long time oenology has abused sulfitation to prevent the oxidative evolution of wine: the experience gained on this practice has had to progressively adapt to the sensory disadvantages caused by an excessive presence of sulfites and the disaffection of consumers for these wines. At the same time, we are witnessing a growing demand for wines in the market with a dominant fruity character, whose aromatic features are most likely associated with oxidation phenomena caused by oxygen levels dissolved in wine that are too high when bottled. In order to be able to produce wines with a low content of sulfites and at the same time with limited concentrations of oxygen it will be necessary to check and evaluate critical enrichment in oxygen in the winemaking process, from receiving grapes to the bottling of wine. During wine aging the balance between sulfites, oxygen, and other aging variables is a kind of unique know-how of the winery, which is based on empirical observations, tasting and tradition, and the difficulty of this art is related to the impossibility to counteract overcompensation, with a possible loss of quality and production of the winery.

The purpose of this work has been to approach this issue from a scientific and technological point of view, with the aim of supporting these operations with quantitative tools based on measurement and modeling. The presence of temperature gradients within the must/wine mass especially during fermentation makes the whole procedure a non-homogeneous and highly dynamical process where the distribution of oxygen plays a key role per se, directly affecting the organoleptic characteristics of wine, and for the evolution of the other quantities involved in vinification, having interactions with sulfites and yeast. With this work we demonstrate the possibility to use specific sensors to continuously monitor the concentration of oxygen in space and time opening the pathway to process prediction and control. This approach becomes an absolutely necessary instrument to optimize the wine process and to obtain a higher quality product and at the same time facilitate the correct dosage of $SO_{2}$ before bottling.

The designed sensor is based on actual lifetime measurement of the PtTFPP/PSF luminescence when excited with specifically tuned LEDs. Measurements based on lifetime are more robust and reproducible than those based on luminescence intensity, since they are not conditioned by many external factors. For example, emission depends on the membrane geometry and a little variation of the membrane position or its degradation can produce important signal intensity variations; conversely, the lifetime remains unaffected. In particular, the developed optical setup and electronic topology allows the measurement of the lifetime transient by means of a low cost Silicon Photodiode, while the PtTFPP/PSF membrane is supported on Mylar^®^. The sensor shows good sensitivity with a maximum theoretical SNR of 34.5 dB resulting in a $R^{2}$ of 0.97 when fitting of the exponential photoluminescence decay. Calibration of the membrane excludes the presence of any interfering species in wine and indicates that ethanol has the effect of reducing the calibration sensitivity, but does not modify the membrane structure. Consistently, the results from the in situ measurement during the aging phase of winemaking confirm the possibility of employment the sensor system as an effective and practical tool to monitor oxygen levels: we can conclude that the system allows to achieve the crucial determination of the concentrations of $O_{2}$ in wine below 10–40 μg/L at 20 ${}^{\circ }$C (saturation of $O_{2}$ in wine at 20 ${}^{\circ }$C and 1 bar is 8.4 mg/L), therefore between 0.1 and 0.5% of $O_{2}$.

On the whole, the experience from the project shows the potential advantages of employing such methodologies and tools during wine production, in particular with respect to:

- the possibility of obtaining a complete and correct fermentation process;
- the reduction of micro-organisms growth risk, since wine naturally contains a microbial load that can proliferate with oxygen;
- the production of a wine with optimized sensory characteristics, namely stabilized color and structure of red wines and stabilization of the aromatic and organoleptic profile;
- the reduction of the perception of dryness and astringency due to the tannic structure;
- less need for antioxidants, such as ascorbic acid and sulfur dioxide, generally added for preservation;
- the increase of the “shelf life” of wine.

Given these aspects, we conclude by observing that although the project has been designed and developed for applications in the wine sector, however, the approach can have wide and natural development targeting other products and needs in the agri-food sector, among which we name the process control in fermentation procedures and environments in controlled or modified atmosphere, the verification of the packaging, both in the packaging phase and in the monitoring of the state of conservation of a food, the indirect measurement of compounds that can react enzymatically with oxygen consumption, and the process control in the field of aeroponic or hydroponic cultures.
